# Crystal optics simulations for delineation of the three-dimensional cellular nuclear distribution using analyzer-based refraction-contrast computed tomography

**DOI:** 10.1038/s41598-022-24249-8

**Published:** 2022-11-15

**Authors:** Naoki Sunaguchi, Zhuoran Huang, Daisuke Shimao, Shu Ichihara, Rieko Nishimura, Akari Iwakoshi, Tetsuya Yuasa, Masami Ando

**Affiliations:** 1 Department of Radiological and Medical Laboratory Sciences, Nagoya University Graduate School of Medicine, Nagoya, Japan; 2 Department of Radiological Technology, Hokkaido University of Science, Sapporo, Japan; 3 Department of Pathology, Clinical Research Center, Nagoya Medical Center, Nagoya, Japan; 4 Graduate School of Engineering and Science, Yamagata University, Yonezawa, Japan; 5 High Energy Accelerator Research Organization, Tsukuba, Japan

**Keywords:** Breast cancer, Cancer imaging, Imaging, Biomedical engineering, Computational science, Scientific data

## Abstract

Refraction-contrast computed tomography (RCT) using a refractive angle analyzer of Si perfect crystal can reconstruct the three-dimensional structure of biological soft tissue with contrast comparable to that of stained two-dimensional pathological images. However, the blurring of X-ray beam by the analyzer has prevented improvement of the spatial resolution of RCT, and the currently possible observation of tissue structure at a scale of approximately 20 µm provides only limited medical information. As in pathology, to differentiate between benign and malignant forms of cancer, it is necessary to observe the distribution of the cell nucleus, which is approximately 5–10 µm in diameter. In this study, based on the X-ray dynamical diffraction theory using the Takagi–Taupin equation, which calculates the propagation of X-ray energy in crystals, an analyzer crystal optical system depicting the distribution of cell nuclei was investigated by RCT imaging simulation experiments in terms of the thickness of the Laue-case analyzer, the camera pixel size and the difference in spatial resolution between the Bragg-case and Laue-case analyzers.

## Introduction

Analyzer-based refraction-contrast X-ray imaging, a type of synchrotron radiation X-ray imaging, converts the angular deviation of X-ray beam as they propagate through an object to a refraction contrast, using a rocking curve near the Bragg condition of a refraction angle analyzer made of Si single crystals installed behind an object^1, 2^. The refractive index in the X-ray region is usually a complex number and is represented by the physical quantity n = 1 − δ + iβ, where i is the imaginary unit and δ and β are phase shift and absorption terms, respectively. When X-ray beams propagate across the boundary between media, they refract according to Snell's law if there is a difference in the real part 1 − δ between the two media. The δ of materials having a low atomic number in the hard X-ray region is proportional to the electron density and is approximately 10^−7^. Although it is difficult to directly observe the angular deviation of X-ray beam produced by such materials in space, the rocking curve of the analyzer has a slope sufficiently sharp to indirectly analyze small angular deviations.

In X-ray diffraction, there are two types of crystal configuration: the Bragg case, in which diffraction waves emerge at the incident surface of the crystal, and the Laue case, in which X-ray beam propagate inside the crystal and diffraction waves emerge at the backside of the crystal. In analyzer-based refraction-contrast X-ray imaging, there exists diffraction-enhanced imaging^1^ adopting the Bragg-case angle analyzer (BAA) and X-ray dark-field imaging adopting the Laue-case angle analyzer (LAA)^3^. Refraction-contrast computed tomography (RCT) based on these methods is used as an observational tool for microanatomy and 3D pathology because of its strong ability to depict biological soft tissue^4–11^.

Both the BAA and LAA are known to spread diffracted beams through repeated diffraction due to the huge number of diffraction planes in the crystal when an X-ray beam enters the crystal^12, 13^. This leads to blurring of the x-ray imaging, which in turn reduces the spatial resolution. In previous studies, simulation experiments based on X-ray dynamical diffraction theory^12, 13^, which describes X-ray propagation in crystals, revealed the spatial resolution of crystal optics^14–17^. The studies showed that the spatial resolution of the BAA remains almost the same from a thickness of a few tens of micron to infinite thickness, whereas the spatial resolution of the LAA is highly dependent on thickness, with a thinner LAA having higher spatial resolution. Additionally, the distribution of X-ray blur in crystal optics is not symmetrical and includes an oscillating component. Meanwhile, the computer performance at the time of the cited study was barely sufficient to evaluate the spatial resolution of projected images using simple absorbing or phase objects, and no evaluation has ever been conducted that went as far as the spatial or density resolution of RCT. Currently, computer performance has improved dramatically, and supercomputers with several PFLOPS using large-scale GPU clusters are readily available, enabling detailed CT simulations that were previously difficult to perform. X-ray image detector performance and Si crystal thinning techniques are also improving, and the image quality of RCT is approaching that of pathological images stained by Hematoxylin–Eosin^9^. Therefore, it is very important to provide the performance limitations of RCT in this study. Especially in diagnostic pathology, because information on the distribution of cell nuclei plays an important role in differentiating cancer from benign lesions, it is clinically significant to investigate the conditions under which RCT can depict the distribution of cell nuclei.

In this study, we performed RCT imaging simulation experiments based on X-ray dynamical diffraction theory of LAA and BAA using the Takagi–Taupin equation, which can be used to calculate the propagation of X-ray energy in a crystal, and investigated crystal optics for RCT, which is necessary for evaluation of the spatial and density resolutions and for delineation of the three-dimensional cell nucleus distribution.

## Results

### Evaluation results of spatial resolution

**Figure 1:**
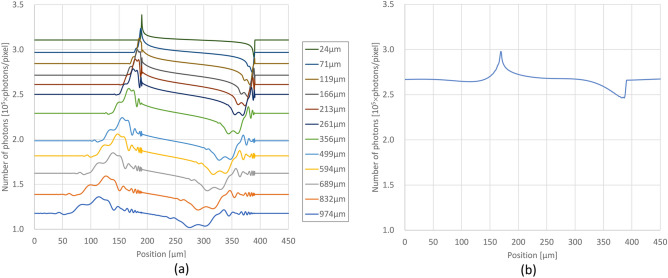
(**a**) Projection profiles for a circular sample with 200 μm diameter at different thicknesses of LAA. (**b**) Projection profile of the same sample taken with BAA. Each graph is plotted at 0.25 μm intervals, with the vertical axis representing the number of photons in the 0.25 μm range. The photon number of incident X-rays is approximately 10^9^ photons/mm.

Before showing the CT results, we first show the X-ray intensity distribution in the emitting plane of the LAA obtained by the simulator, i.e., the projection image. Figure 1a shows 1D projection profiles of a circular sample with a diameter of 200 μm taken under different LAA thicknesses and a pixel size of 0.25 μm, which is plotted at pixel size intervals, with the vertical axis representing the number of photons incident on one pixel. The X-ray beam enters the crystal from a direction that is off from the Bragg angle by approximately 0.5 angular seconds. The photon number of incident X-rays is approximately 10^9^ photons/mm. The profiles at each thickness are blurred to the left as the LAA thickness increases as in previous studies^14, 15, 17^ that investigated the spatial resolution of the projection image, which indicates that the simulator is working properly. Figure 1b shows the projection profile of the same sample taken with BAA. The BAA profile is a smooth curve without the oscillations that appeared in the LAA, while the refractive component of circular sample can be seen flowing in one direction from right to left as in the LAA above 119 μm thickness.

**Figure 2:**
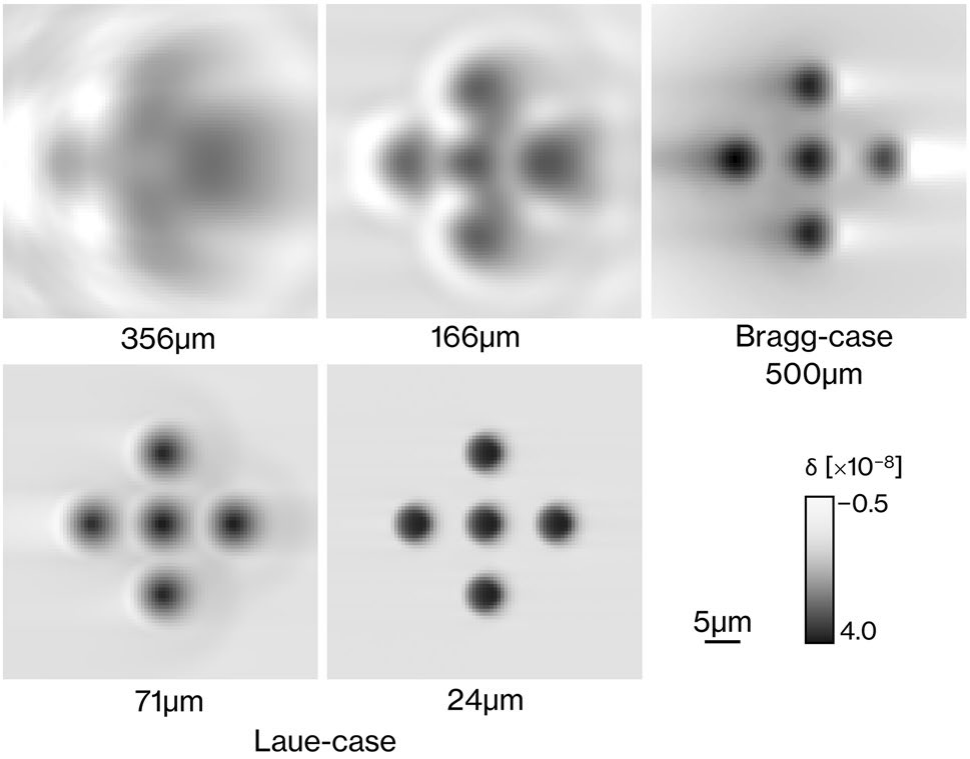
The reconstructed images of a numerical phantom consisting of a circle with a diameter of 5 µm, imaged by LAA with different thicknesses and BAA with 500 μm thickness.

**Figure 3:**
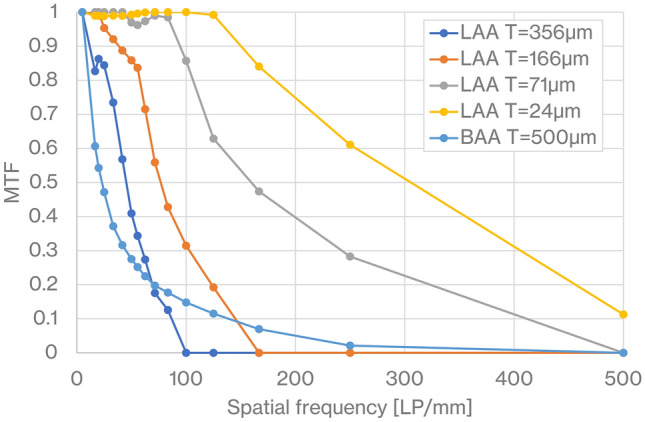
MTFs at different spatial frequencies, calculated from a line profile across the center of three circles arranged horizontally on the CT image of the phantom shown in Fig. 2.

Figure 2 shows the reconstructed RCT images of a numerical phantom consisting of a circle with a diameter of 5 µm, imaged by LAA with different thicknesses and BAA with 500 μm thickness. The pixel size is 0.5 μm. CTs are reconstructed from a projection obtained within an angular range around 180° of the sample, but the reconstructed image has asymmetric contrast because the refractive component of the projection flows in one direction from right to left as shown in Fig. 1. LAA images show that as the thickness of the LAA decreases, the blur decreases. The sharpness of the circles on the BAA image is close to that of the LAA with 71 μm thickness, but strong artifacts extend to the left and right of the circle. Figure 3 shows modulation transfer functions (MTFs) calculated from the RCT images of each numerical phantom, imaged by LAA with different thicknesses and BAA with 500 μm thickness. MTF for each spatial frequency improves with decreasing thickness of the LAA, where the variation in the MTF for the LAA seen in the range of 0–100 line pairs (LP)/mm is due to the nonuniformity around the circle in RCT, which results from the spatial fringe of X-ray beam in the crystal. The MTF of the BAA drops sharply to a low spatial frequency of approximately 50 LP/mm and declines slowly thereafter. At 100 LP/mm at 356 µm thickness of LAA, 166 LP/mm at 166 µm thickness of LAA, and 500 LP/mm at 71 µm thickness of LAA and 500 µm thickness of BAA, where the value of MTF is zero, adjacent circles cannot be resolved by visual inspection of the RCT image.

**Figure 4:**
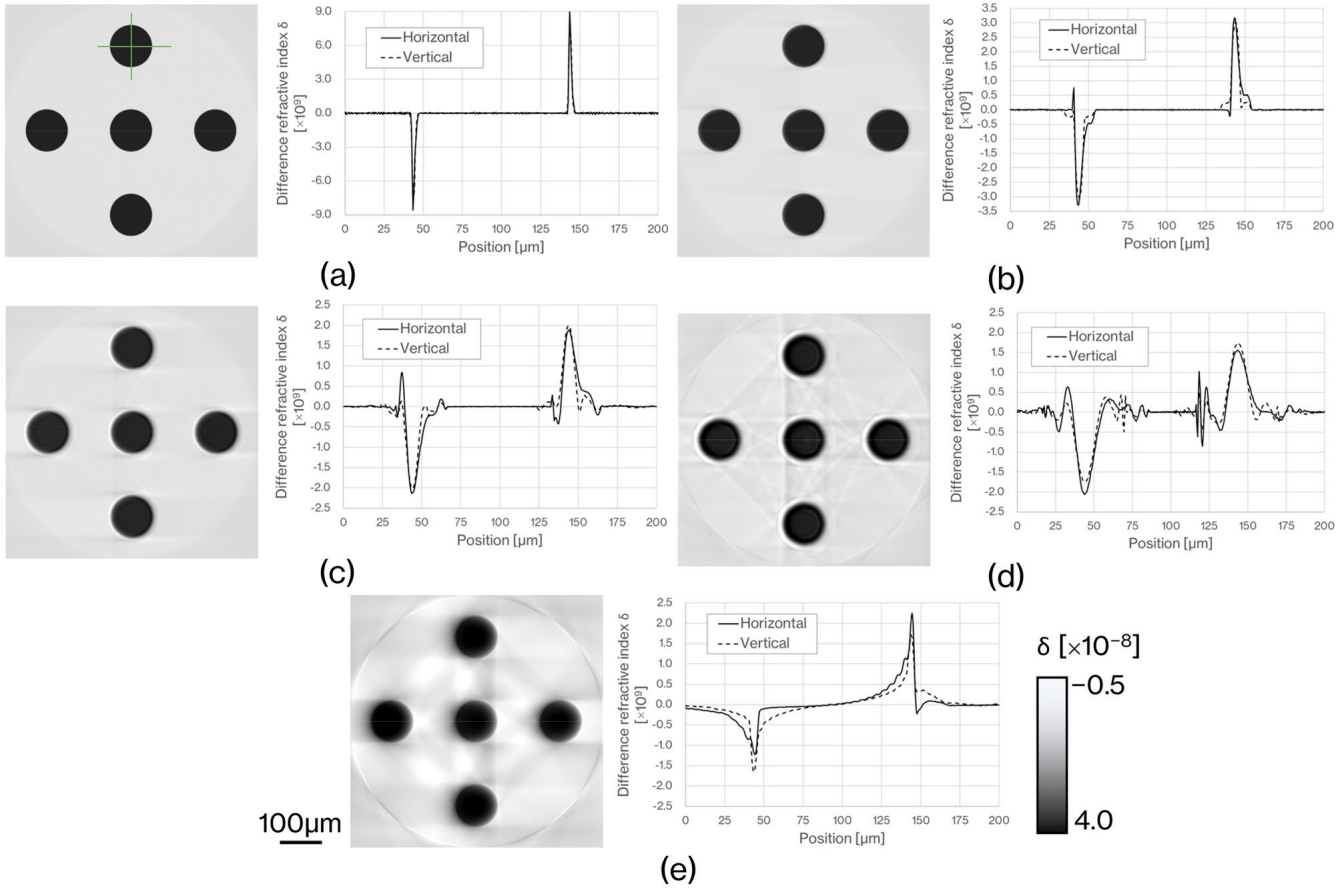
Reconstructed images and LSFs in the horizontal and vertical directions of the numerical phantom comprising circles having a diameter of 100 µm. LSFs were constructed by differentiating the line profile (green line) of the image by each thickness condition. (**a**) LAA thickness of 24 µm, (**b**) LAA thickness of 71 µm, (**c**) LAA thickness of 166 µm, (**d**) LAA thickness of 356 µm, and (**e**) BAA thickness of 500 µm.

Figure 4 shows reconstructed images of a numerical phantom comprising circles with a diameter of 100 µm and line spread functions (LSFs) calculated from the line profiles of the circle edges (indicated by green lines). The pixel size on each CT image is 0.5 µm. Figure 4a–e respectively show the results for LAAs with thicknesses of 24, 71, 166, and 356 µm and a BAA with a thickness of 500 µm. It is seen that as the thickness of the LAA decreases, the image becomes clearer and the streak-like artifacts at the edge of a circle diminish. The RCT image obtained by the BAA shows a band artifact extending horizontally from the circle as in Fig. 2. The LSF of the LAA generated sharper peaks at the edges for thinner LAAs. This implies that the image obtained by a thinner LAA is less blurry. It is also seen that as the LAA becomes thicker, there are more vibrations near the edges, which are inherently flat. The LSF of the BAA shows no appreciable vibrations, but is narrower vertically than horizontally. Table 1 summarizes the full widths at half maximum (FWHMs) of the LSFs in Fig. 4. The LSFs in Fig. 4 are obtained on both sides of the edge, and each value in Table 1 is an average value for the two FWHMs. The thinner the LAA, the smaller the FWHM. The FWHM of the BAA is slightly narrower than that of the LAA with a thickness of 166 µm.

**Table 1:** FWHMs of the LSF in the horizontal and vertical directions of the circular structure in the numerical phantom shown in Fig. 4.

Analyzer type	Bragg	Laue			
Crystal thickness (µm)	500	356	166	71	24
Horizontal (µm)	7.16	11.60	8.23	4.60	1.78
Vertical (µm)	4.65	11.67	7.73	4.97	2.23
Mean (µm)	5.91	11.64	7.98	4.79	2.01

### Evaluation results of the density resolution

**Figure 5:**
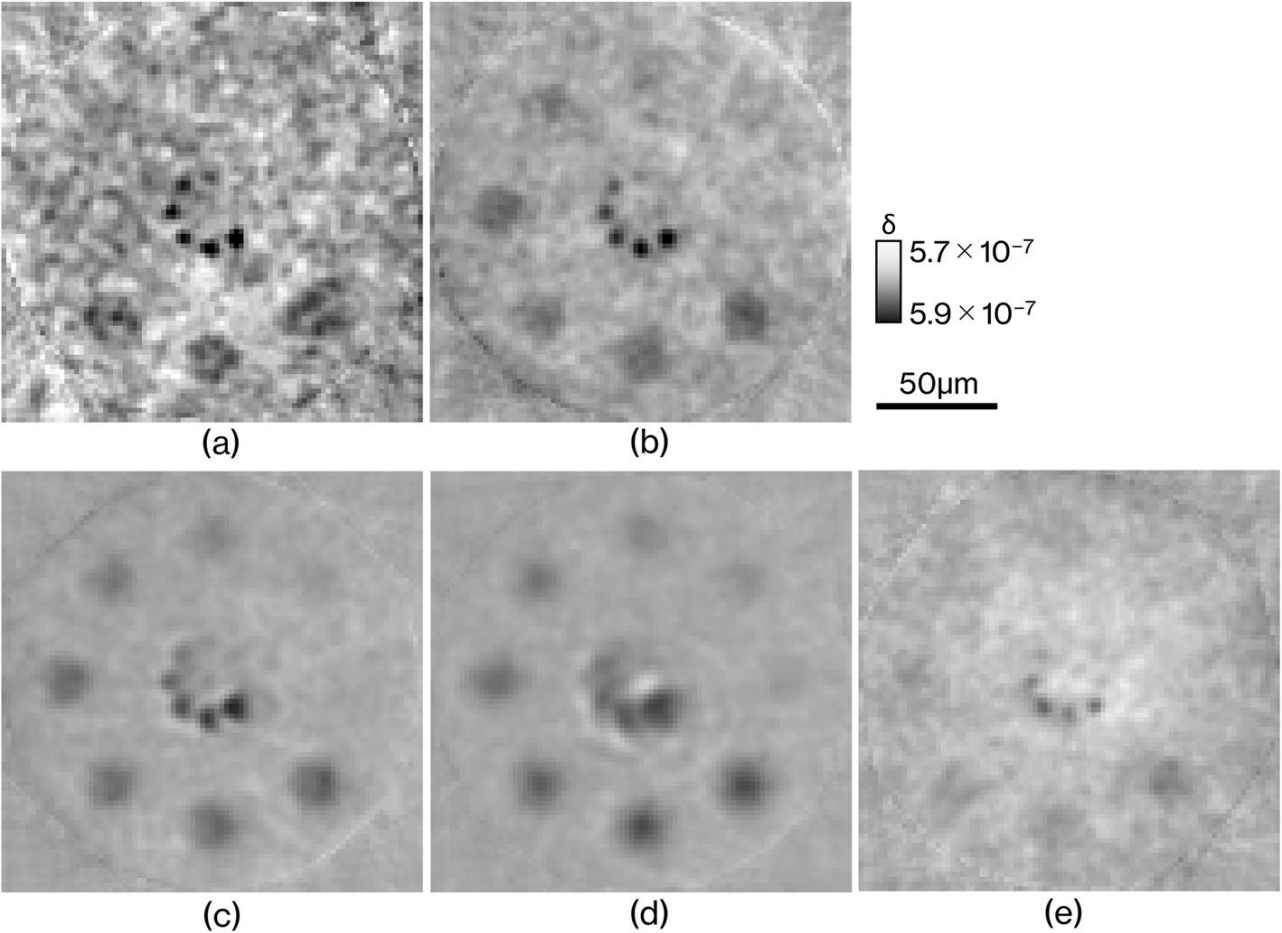
Reconstructed images of the numerical phantom for density resolution measurement. (**a**) LAA thickness of 24 µm, (**b**) LAA thickness of 71 µm, (**c**) LAA thickness of 166 µm, (**d**) LAA thickness of 356 µm, and (**e**) BAA thickness of 500 µm.

Figure 5 shows reconstructed RCT images of the phantom for density resolution evaluation. The pixel size on each CT image is 2 µm. Figure 5a–d show the results for LAAs with thicknesses of 24, 71, 166, and 356 µm and (e) shows the results for the BAA with thickness of 500 µm. The circle structures of the reconstructed images are easily buried in noise because the relative density with water set in a circle becomes smaller from the direction of *h* to *a* in Fig. 10. As the LAA thickness deceases, the 5-µm-diameter circle becomes sharper, although the noise becomes stronger; in Fig. 5d, the shape of the 5-µm circle is almost completely lost. The main reason why noise becomes stronger as crystals become thinner is that high-frequency noise components are emphasized due to the improved sharpness of the image. On the other hand, the thinner the crystal, the lower the X-ray absorption by the crystal and the higher the S/N of the image, but the difference in attenuation between 24 µm and 356 µm is only approximately 28%, which has almost no effect on image quality. A circle with a diameter of 5 µm and a density of 1% can be seen under the 24-µm (Fig. 5a) and 71-µm (Fig. 5b) thickness conditions. Meanwhile, 20-µm circles are more easily seen with the thicker LAA. The BAA image in Fig. 5e delineates a circle of 5-µm diameter and 2% density, but the contrast of the 20-µm diameter circle is lower than the LAA image, and appears to have an image quality to be between 71-µm (Fig. 5b) and 166-µm (Fig. 5c) LAAs. Contrast to noise ratios (CNRs) defined by equation (3) calculated for each circle having a diameter of 5 µm and density of 3% in Fig. 5a–e are 4.13, 6.91, 9.80, 15.96, and 5.69, respectively.

### Evaluation results of the descriptive ability for the cell nucleus distribution

**Figure 6:**
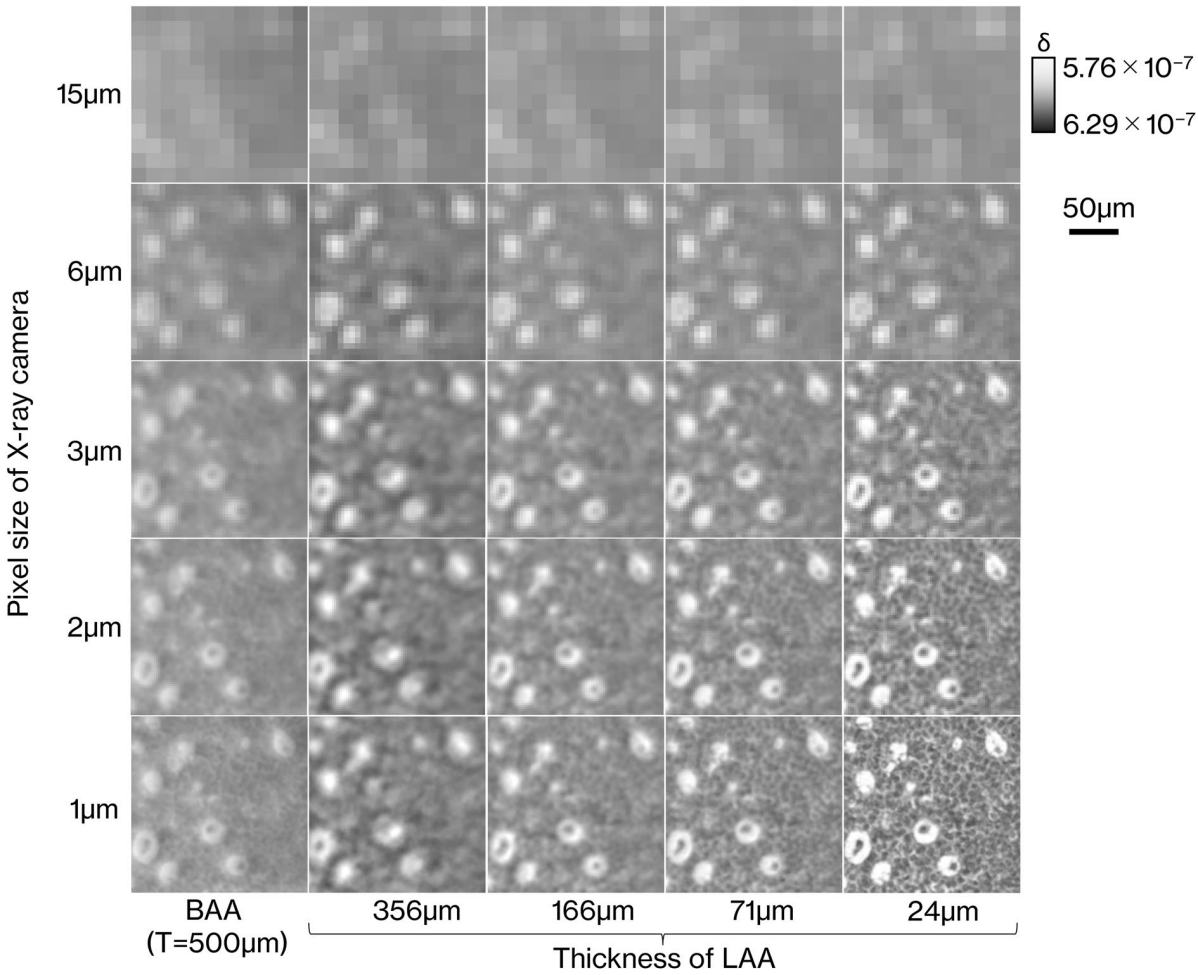
Reconstructed images of numerical phantoms converted from a breast cancer tissue specimen for evaluating the ability to delineate the cellular nuclear distribution. Each image is a magnified image of the area within the yellow square in Fig. 10a.

Figure 6 is a magnified image of the orange square in the upper left image of Fig. 11 from the reconstructed image of the phantom for each pixel size and LAA with different thicknesses or BAA with 500 μm thickness. Pixel sizes tested were 1, 2, 3, 6, and 15 µm. The distribution of the glandular lumen is clearly delineated in all images with pixel sizes smaller than 6 µm. When the pixel size reaches 3 µm, the contents of the glandular lumen can be seen. The distribution of cell nuclei is clearly depicted at a pixel size of 1 µm and LAA thickness of 24 µm, but a rough distribution can also be seen at a pixel size of 1 µm and LAA thickness of 71 µm or a pixel size of 2 µm and LAA thickness of 24 µm. The descriptive ability of BAA appears to be between 71 µm LAA and 166 µm LAA, as the results obtained for the density resolution evaluation.

## Discussion

Figures 3 and 4 show that the spatial resolution achievable with a thinner LAA is approximately 2 µm, which is smaller than the cell nucleus. The results in Fig. 5 show that the density resolution is sufficient to obtain the density difference between the cell nucleus and cytoplasm at any thickness of the LAA. Figure 6 shows the clear depiction of the distribution of cell nuclei under the conditions of a pixel size of 1 µm and LAA thickness of 24 µm. We here discuss the overall spatial resolution of the imaging system, further improvement of image quality, the difference between BAA and LAA, and pathological applications.

The overall spatial resolution of an imaging system is related to the focus size of X-ray source, the imaging geometry, the analyzer, and the X-ray detector. In this study, we ignored the blur caused by the focus size and imaging geometry, and considered only the blur caused by the analyzer and the pixel size of the X-ray detector. The incident beam in the simulation is a perfect plane wave, and the distance between the object and the detector does not affect the spatial resolution. However, in actual measurements using synchrotron radiation beamlines, the distances between the object and the detector or between the object and the crystal must also be considered. The amount of blur due to focus size and imaging geometry can be calculated as standard deviation of the point spread function (PSF) on the sample, σs = σ × Ld/Ls, where σ, Ls, and Ld represents the focus size of X-ray source with Gaussian intensity distribution, the geometric focus-to-object distance, and the object-to-sensing surface distance. The spatial resolution can thus be improved by moving the detection plane closer to the object. As an example, when using the BL14B beamline (vertical focus size of 72 µm, distance from focus to object of 25 m) of KEK-PF, the Japanese synchrotron radiation facility, to obtain a spatial resolution of 1 µm in the vertical direction, the distance between the object and detector should be 0.347 m. However, the use of the analyzer requires a certain amount of working space because the analyzer crystal is inserted between the object and detector. The global point spread function representing the blur in the measurement system (PSFglobal) is the convolution among the point spread functions of the geometry, of the analyzer, and of the X-ray detector (PSFgeometry, PSFanalyzer, and PSFdetector, respectively), that is, PSFglobal = PSFgeometry * PSFanalyzer * PSFdetector, where * represents the convolution product. The X-ray blur caused by the analyzer occurs in the one-dimensional direction as shown in Fig. 1, is more dominant than the other blur factors in the measurement. To further improve spatial resolution, a small Bragg angle can be used to reduce the angular divergence of X-ray beam propagating in the crystal. For example, by increasing the incident X-ray energy from 20 keV (used in this simulation) to 60 keV, the Bragg angle of Si(111) is reduced from 5.673 to 1.888° by a third. Note that the image contrast is reduced because the interaction between the X-ray and the object is smaller.

BAA is commonly chosen over LAA in analyzer-based imaging because it is thought to provide higher spatial resolution than LAA and the spatial resolution is independent of BAA thickness. On the other hand, as shown in Figs. 5 and 6, the image quality of the BAA in this study is between the LAA with thicknesses of 71 and 166 µm, a result that differs from previous knowledge. In the MTF results in Fig. 3, the trend of the curve differs significantly between BAA and LAA, with BAA having a lower MTF in the low-frequency region than LAA, which may be linked to the fact that some 20 µm diameter circles are not depicted in Fig. 5e. Another reason BAA is selected is that it has a thick block shape and can be installed stably, but the Bragg angle utilized is usually acute, making it difficult to be shortened the distance between the sample and the camera to improve spatial resolution. Although a thin LAA requires a technique to install it without distortion, it easily allows to keep a short distance between the sample and the camera because it can be placed almost parallel to the detection plane of the camera.

The results of the density resolution evaluation shown in Fig. 5 suggest that the size of the cell nucleus can be delineated by making the LAA crystals thinner, but the noise in the image increases, which may interfere with distinguishing between the noise and signal. Although the conditions of this simulation are based on an actual experiment performed on the BL14B beamline of KEK-PF, the noise can be reduced by increasing the exposure amount, adopting image processing such as machine learning, and using a high-brilliance fourth-generation synchrotron radiation facility.

The image with a pixel size of 1 µm and a LAA thickness of 24 µm in Fig. 6 not only clearly depicted the distribution of the cell nuclei, but also showed the cell nuclei surrounding the glandular lumen, which is one of characteristic growth pattern of ductal carcinoma in situ of the breast. This indicates the possibility that RCT can be used to differentiate cancer from benign lesions as in diagnostic pathology. Also, microscopes used in pathological diagnosis generally provide information of cancer only in a two-dimensional plane, whereas RCT can follow the spread of cancer in three dimensions. Furthermore, although ductal carcinoma in situ of the breast, which still reside in its normal place progress to invasive carcinoma, which is potentially lethal, at some point, three-dimensional observation of the boundary between non-invasive and invasive carcinoma may allow us to learn about unknown cancer mechanisms.

## Methods

### Generation of refraction-contrast images based on X-ray dynamical diffraction theory

**Figure 7:**
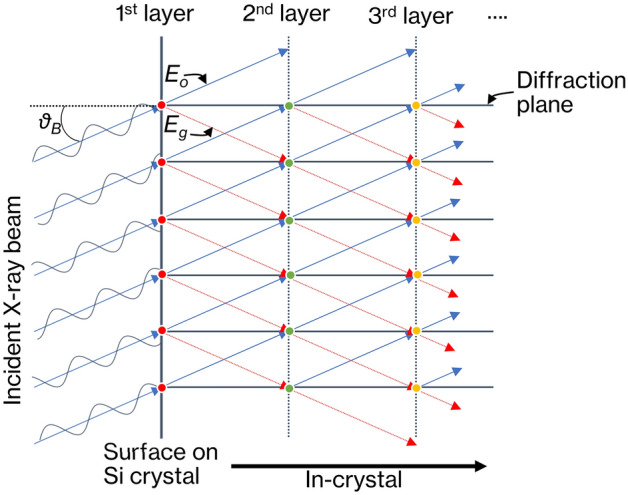
X-ray beam propagation in the LAA.

The Takagi–Taupin equation describes X-ray wave propagation based on X-ray dynamical diffraction theory in Si single crystals:

1 $$\frac{\partial {E}_{o}}{\partial {S}_{o}}=\frac{1}{2}K{\chi }_{o}^{{{\prime\prime}}}{E}_{o}-\frac{i}{2}KP{\chi }_{\overline{g}}{E }_{g},\quad \frac{\partial {E}_{g}}{\partial {S}_{g}}=-\frac{i}{2}KP{\chi }_{g}{E}_{o}+\frac{1}{2}K{\chi }_{o}^{{^{\prime}}{^{\prime}}}{E}_{g},$$

where *E*_*o*_ and *E*_*g*_ are the complex amplitudes of X-ray beam propagating inside the crystal, expressed by making two-wave approximations in the forward and diffraction directions, respectively. *S*_*o*_ and *S*_*g*_ respectively denote the positions in the forward and diffraction directions. *χ*_*o*_ is the Fourier coefficient of the zeroth order of electrosensitivity, and *χ*_*o*_'' is the imaginary part of *χ*_*o*_. *χ*_*g*_ is the Fourier coefficient of the *g*th order of electrosensitivity. ${\chi }_{\overline{g} }$ is the complex conjugate of *χ*_*g*_. *K* and *P* are respectively the wavenumber and polarization factor for vacuum. Although the Takagi–Taupin equation can include the contribution of the crystal strain field, the experiment assumes that there is no distortion in the crystal. When thin crystals are used in actual experiments, ideas such as polishing only near the center while maintaining the peripheral thickness of the crystal are needed. The differential equations are analyzed using the Euler method. A comparison of images obtained by the simulation with those obtained in actual experiments reveals that the calculation error in adopting the Euler method does not affect the analysis. The calculation of the wave propagation of X-ray beam within the crystal begins at the point where the X-ray beam enters the surface of the Si single crystal. Figure 7 shows an example of X-ray wave propagation in the LAA, where *θ*_*B*_ is the Bragg angle of the crystal. When the wavefront of the X-ray beam is incident on the Si single-crystal surface (first layer) from a direction close to the Bragg angle, which is the diffraction condition of Si single crystals, the incident wave is divided into *E*_*o*_ and *E*_*g*_ in the *S*_*o*_ and *S*_*g*_ directions according to Eq. (1) at each diffraction plane (red points). At the diffraction plane of the second layer (green point), *E*_*o*_ and *E*_*g*_ generated in the first layer merge and again split in the *S*_*o*_ and *S*_*g*_ directions according to Eq. (1). This calculation is performed up to the crystal backside in layer order. The X-ray beam incident on the LAA spreads and propagates over a range of 2*θ*_*B*_ which is the angle between *E*_*o*_ and *E*_*g*_ directions, and the diffracted X-ray beam on the backside of the LAA is thus wider than the incident beam. Therefore, the magnitude of the X-ray blur is proportional to the thickness of the crystal. The initial values of *E*_*o*_ and *E*_*g*_ at each diffraction plane in the first layer (*S*_*o*_ = 0, *S*_*g*_ = 0), immediately before X-ray incidence on the crystal, are given as *E*_*o*_ = exp(*iφ*), *E*_*g*_ = 0, where *φ* is the X-ray phase, which is determined by the X-ray wavefront emitted from the numerical phantom. An X-ray image detector with ideal virtual pixels which does not consider image quality degradation due to the thickness of a scintillator or electrical noise is placed behind the crystal to obtain refraction contrast images. The flow of data generation at the detector is as follows. First, the simulation of X-ray wave propagation in the crystal outputs *E*_*o*_ and *E*_*g*_ for each diffraction plane. Second, after integrating all *E*_*o*_ reaching the detector pixel by pixel, the power meaning the X-ray intensity is calculated from the complex number of each pixel. Third, for each pixel, the intensity is converted to a photon count close to the actual experimental condition. Finally, quantum noise having a Poisson distribution is added to the number of photons per pixel. In the BAA simulation, X-ray beam are incident on the surface of the diffraction plane and *E*_*g*_ diffracted at the surface of the crystal is outputted. Propagation within the crystal is the same as in LAA simulation.

### Overall view of the RCT imaging simulator

**Figure 8:**
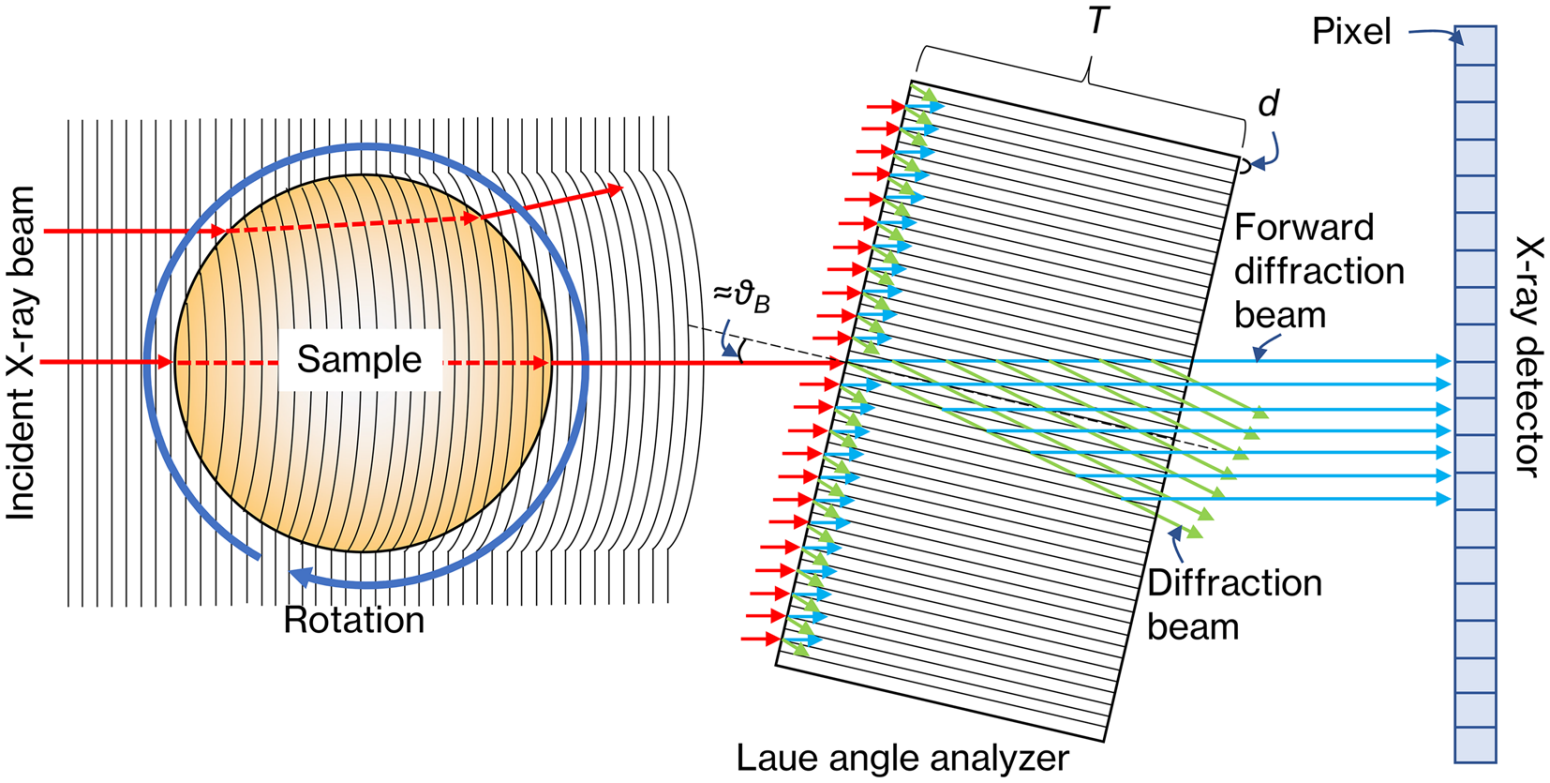
Overall view of the RCT imaging simulator.

Figure 8 shows the overall view of the RCT imaging simulator. Incident X-ray beam, which are plane waves, are first incident on a numerical phantom set in space. In the actual experiment, the sample is rotated in a water tank to reduce unnecessary refraction at the sample surface, and the numerical phantom is thus inserted similarly into a rectangular water volume in this simulation. The plane wave is phase shifted according to the refractive index distribution in the phantom and enters the crystal from a direction that is off from the Bragg angle by approximately 0.5 angular seconds. A refraction contrast image is obtained through the above numerical calculation. Rotation of the phantom and image acquisition are repeated from 0° to 180° to obtain the projection set needed for RCT reconstruction. The RCT reconstruction algorithm uses a filtered backprojection method based on the refraction contrast^18^. Since these calculations are time consuming, they are performed in parallel using the Nagoya University supercomputer Furo Type II system (GPU: up to 6.895 PFLOPS). To obtain a single CT data set of the numerical phantom in Fig. 11 with a crystal thickness of 500 μm, the computation time takes 1800 h on a workstation with single GPU device (NVIDIA Quadro GV100), whereas it takes 2 h at the earliest on a supercomputer.

### Numerical phantom for evaluating spatial resolution

**Figure 9:**
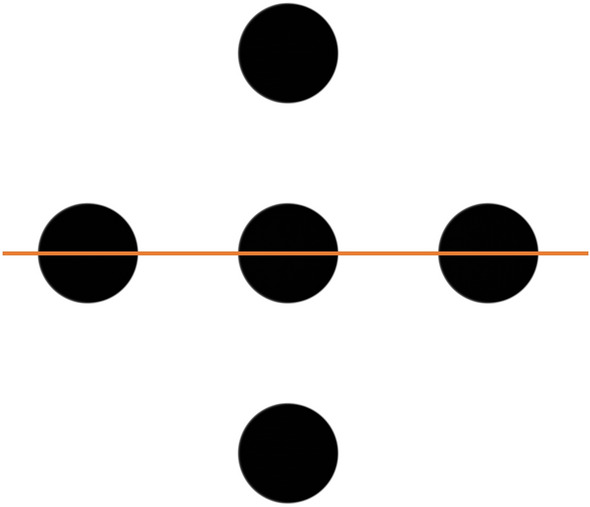
Numerical phantom comprising five circles having different diameters. The diameter of a circle and the distance between adjacent circles are equal.

Figure 9 is a schematic diagram of the numerical phantom for evaluating spatial resolution. Five circles are arranged in a crisscross pattern. The diameter of a circle and the distance between adjacent circles are equal. The δ of each circle is set at 6.1 × 10^−7^ (20 keV), which is close to that of biological soft tissue. RCT of 16 different phantoms with varying circle diameters ranging 1–100 µm were measured, and the spatial resolution was evaluated according to whether adjacent circles could be resolved.

### Numerical phantoms for evaluating density resolution

**Figure 10:**
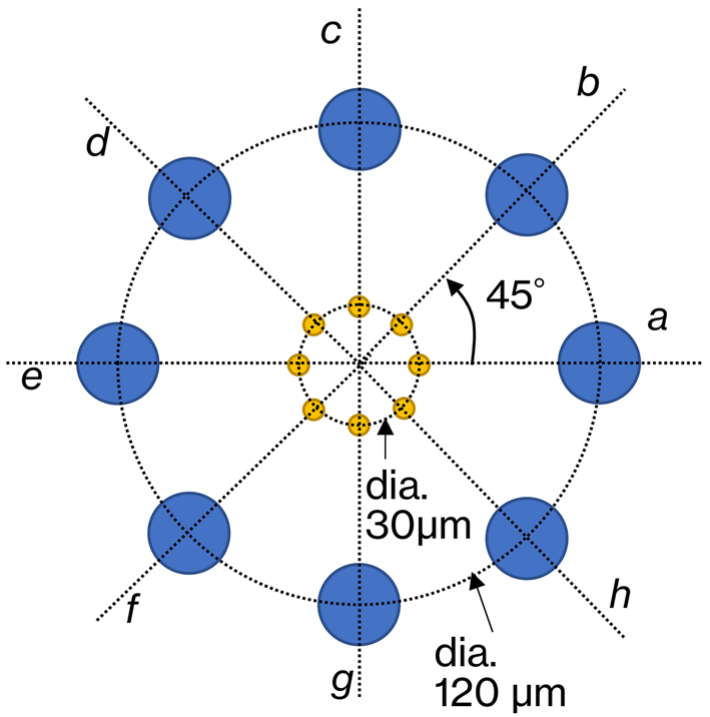
Numerical phantom for density resolution measurement in which eight circles of 5 µm diameter in concentric circles of 30 µm in diameter (drawn as yellow circles) and eight circles of 20 µm diameter in concentric circles of 120 µm in diameter (drawn as blue circles) are arranged at 45° intervals. Symbols a–h represent directions from the center of the phantom.

Figure 10 shows an overview of the numerical phantom for evaluating density resolution. This phantom comprises eight circles each with a diameter of 5 µm and eight circles each with a diameter of 20 µm. The diameters correspond to the size of the cell nucleus and glandular lumen of the organism as observed in a pathology image, and each circle is set to a different electron density that is close to the electron density of water. Table 2 gives the electron density set for each circle, expressed relative to the density of water. The density difference between the cell nucleus and cytoplasm is known to be approximately 3%^19^, and the density difference of the small circle is thus set around 3%. The glandular lumen in living organisms is usually filled with liquid, and the density of the large circle is thus set close to that of water. In general, smaller structures require higher density differences to be observed in computed tomography (CT).

**Table 2:** Electron densities set for circles of 20 and 5 µm diameter of the phantom.

	*a*	*b*	*c*	*d*	*e*	*f*	*g*	*h*
Circles having a diameter of 20 µm	0.1	0.2	0.3	0.4	0.5	0.6	0.7	0.8
Circles having a diameter of 5 µm	0.5	0.8	1.0	2.0	3.0	4.0	5.0	6.0

Values are the difference (+ %) between the electron density of water.

### Numerical phantoms for evaluating the ability to delineate the cellular nuclear distribution

**Figure 11:**
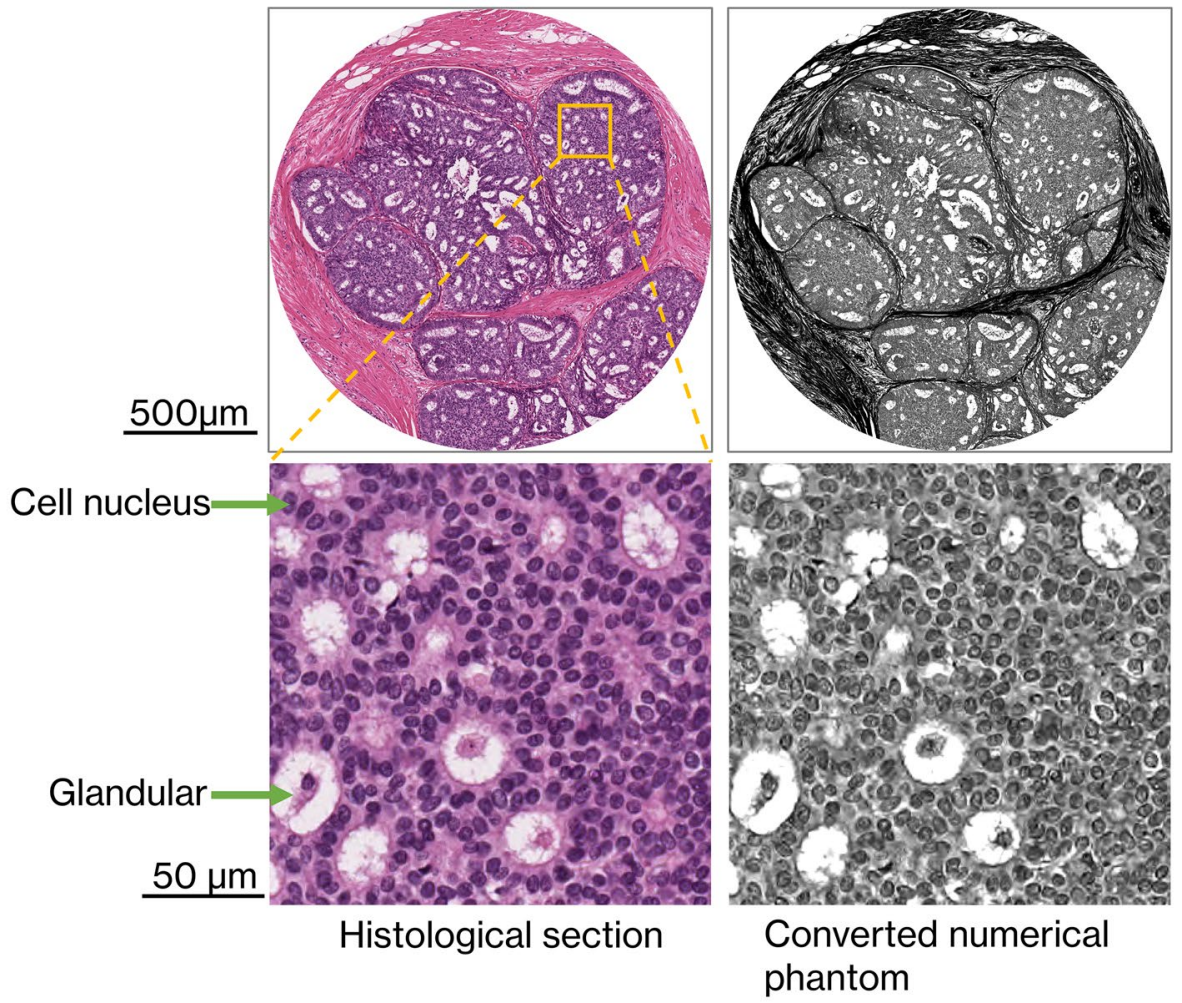
A histological section of a breast tissue specimen stained with hematoxylin and eosin and a numerical phantom image generated from (**a**).

Numerical phantoms are created from an actual pathological image to evaluate the ability to depict the cellular nuclear distribution. The upper left image of Fig. 11 is a pathological image of breast tissue with ductal carcinoma in situ removed from a woman diagnosed with breast cancer. Ductal carcinoma in situ is a non-invasive breast cancer characterized by a proliferation of abnormal epithelial cells confined within the basement membrane and is diagnosed based on gross and histological features included the architectural arrangement or growth pattern of cells, the size and shape of cells and their nuclei viewed under the microscope. The sliced tissue was stained with hematoxylin and eosin and examined under a digital optical microscope at 20× magnification, and an image having a 24-bit color scale and a pixel size of 0.5 µm was then saved. The image has dimensions of 1 mm × 1 mm. The purple-stained structures are the distribution of cell nuclei proliferated by the cancer, and the white cavities are glandular luminal structures caused by cell necrosis or expansion due to secretions. The distribution of the nuclear structure can be clearly seen at this magnification. RCT based on X-ray dark-field imaging has shown in physical experiments that the ratio of mean pixel values in cancer cell tissue, fibrous tissue, and gland luminal regions is approximately 0.45:1:1.45. The pathological image with the color scale is converted to grayscale using an equation that preserves this ratio:

2 $$Y=\left(2.0\times R-1.4\times G-2.0\times B\right)+240$$

where R, G, and B are the red, green, and blue components of a given pixel in the pathological image, each in 8-bit grayscale. Next, each grayscale pixel is converted to a δ. The interior of the glandular lumen is replaced by water, and the mean value of δ for the pixels in the glandular lumen region can thus be estimated to be 5.76 × 10^−7^ (20 keV), which is the value of δ for water. From the above contrast ratio, δ of the cellular and fibrous tissues can be estimated to be 6.00 × 10^−7^ (20 keV) and 6.29 × 10^−7^ (20 keV), respectively, with respect to that of the glandular lumen. Normalizing each pixel so that the mean value of the pixels in the glandular luminal, cellular tissue, and fiber tissue regions are these three refractive indices creates a virtual distribution of δ in the pathological image. The upper right image of Fig. 11 is a numerical phantom converted from the pathological image.

### Simulation experiments

Simulation experiments will show the spatial resolution, the density resolution, and whether the distribution of cancer cell nuclei in biological soft tissues can be depicted from RCT using BAA or LAAs having different thicknesses. Three types of experiment are conducted for this purpose. The first is conducted to quantitatively evaluate the spatial resolution of RCT using BAA or LAAs of different thickness. The second is conducted to quantitatively evaluate the density resolution of RCT using BAA or LAAs of different thickness. The third is conducted to evaluate the LAA thickness and camera pixel size that can depict the distribution of cell nuclei from a numerical phantom of a pathological image. The diffraction plane of analyzer crystals under consideration is Si(111). Since the lattice spacing on the Si(111) plane is small compared to the spacing on other planes, a small Bragg angle can be used, which can reduce the angular divergence of X-ray beam within the crystal leading to improved spatial resolution. The LAA thicknesses considered are 24, 71, 166, and 356 µm, which are thickness conditions where the forward diffraction intensity is theoretically zero at Bragg angle incidence. The thickness of the BAA is 500 µm which can obtain a silk-hat shaped smooth rocking curve. Since diffraction by BAA occurs mainly near the surface of the crystal, the thickness of the crystal has little effect on image quality.

### Evaluation experiment for the spatial resolution

The spatial resolution is evaluated by acquiring a horizontal line profile across the center of circles in RCT as shown in Fig. 9 and from the ratio of the minimum and maximum values of the profiles; i.e., the contrast between the circle and non-circle areas. A larger ratio makes it easier to resolve adjacent circles. The imaging system with higher spatial resolution can image phantoms comprising circles with smaller diameters with higher contrast. In this experiment, after obtaining the circle contrast from each CT obtained with different circle diameters for each analyzer and numerical phantom, an MTF is created to represent the spatial frequency characteristics. The spatial frequency is defined as LP/mm, where LP is a set of a circle and a non-circle between two circles on the orange line of Fig. 9. The numerical phantoms have 16 circle diameters: 1, 2, 3, 4, 5, 6, 7, 8, 9, 10, 12, 15, 20, 50, and 100 µm. MTFs are obtained point-by-point from CT images corresponding to each diameter, shown in Fig. 3. MTFs can also be obtained by applying a Fourier transform to LSFs obtained from the edge of a circle, but we have not chosen this method because the region around the circle contains complex oscillations due to multiple diffraction of the crystal. As another way to evaluate the blur on an image, we measure FWHM of LSFs obtained from the edge of a 100 µm diameter circle, shown in Fig. 4. In this experiment, the effects of the number of incident X-ray photons and noise are not considered so as to evaluate only the blurring of the image caused by the analyzer. In addition, the pixel size of the camera is 0.5 µm, which is smaller than the circle diameter.

### Evaluation experiment for the density resolution

The density resolution is evaluated using visual methods and contrast-to-noise ratios for the reconstructed RCT images. The contrast-to-noise ratio is defined by

3 $$CNR=\frac{{m}_{obj}-{m}_{back}}{{\sigma }_{back}},$$

where *m*_*obj*_ and *m*_*back*_ are the mean values of δ of the circle and extra-circle regions in the reconstructed image, respectively. *σ*_*back*_ is the standard deviation of δ of the extra-circle region. The CNR is calculated for each circle of different reconstructed relative density. The pixel size of the camera is 2 µm, which is less than half the diameter of the smaller circle.

### Evaluation experiment for the ability to describe the cell nucleus distribution

The descriptive ability of the cell nucleus distribution is evaluated using visual methods. The imaging conditions for each experiment are summarized in Table 3.

**Table 3:** Imaging conditions for each simulation experiment.

Experiment	Evaluation of the spatial resolution	Evaluation of the electron density resolution	Cell nucleus delineation
X-ray energy (keV)	20		
X-ray photons (photons/mm^2^/s)	Not considered	10^8^	
Exposure time (s)	Not considered	4	
Projection views	720	450	1.5 times the number of pixels of the detector
CT algorithm	Filtered backprojection method with signum and Shepp–Logan filters		
Pixel size (µm)	0.5	2	1, 2, 3, 6, 15
Noise	Not considered	Quantum noise	

## Data Availability

The datasets used and/or analysed during the current study available from the corresponding author on reasonable request.
